# International Classification of Diseases – 11th revision: from design to implementation

**DOI:** 10.11606/s1518-8787.2020054002120

**Published:** 2020-11-04

**Authors:** Manuella Santos Carneiro Almeida, Luis Ferreira de Sousa, Patrícia Moreira Rabello, Bianca Marques Santiago

**Affiliations:** I Universidade Federal da Paraíba Programa de Pós-Graduação em Odontologia Centro de Ciências da Saúde João PessoaPB Brasil Universidade Federal da Paraíba. Programa de Pós-Graduação em Odontologia. Centro de Ciências da Saúde. João Pessoa, PB, Brasil; II Hospital Estadual de Emergência e Trauma Senador Humberto Lucena João PessoaPB Brasil Hospital Estadual de Emergência e Trauma Senador Humberto Lucena. João Pessoa, PB, Brasil; III Centro Odontológico de Estudos e Pesquisas João PessoaPB Brasil Centro Odontológico de Estudos e Pesquisas. João Pessoa, PB, Brasil; IV Universidade Federal da Paraíba Centro de Ciências da Saúde Departamento de Clínica e Odontologia Social João PessoaPB Brasil Universidade Federal da Paraíba. Departamento de Clínica e Odontologia Social. Centro de Ciências da Saúde. João Pessoa, PB, Brasil

**Keywords:** International Classification of Diseases, World Health Organization, Public Health, Epidemiology

## Abstract

The World Health Organization launched in May 2019 the new International Classification of Diseases (ICD), 11th revision. As a contribution to this transition, this article aims to present the main changes of the revised version of the classification and indicate the most pressing challenges. After 30 years of the ICD-10 publication, we identified significant challenges regarding the new classification, which was presented for adoption by several countries and will be in force in January 2022. The purpose of the preview is to allow countries to plan its use and train their professionals. The new version is completely digital, thus reducing notification errors and facilitating the dissemination and consolidation of this new version. The update highlights the advances in scientific understanding, and it demands structural actions and implementation efficiency from governments, so that everyone who deals with assistance can speak the same language, on a global scale.

## INTRODUCTION

Considering the social and economic development established and emphasized by globalization, health actions must find a new focus. The inability of timely and curative care to embrace actions with significant effect on social needs highlights the need for a more complex and planned care vision of public health actions. Thus, it is necessary to anticipate actions in the face of injuries that affect society in order to minimize expenses.

This need for planning prevention and health promotion actions sustains an increasingly reliable and planned data collection. In this context, according to the World Health Organization (WHO)^1^, the International Classification of Diseases (ICD) appears as a fundamental tool to establish public policies aligned with social needs.

ICD supports the identification of statistical health trends worldwide. As the main tool for coding mortality and morbidity problems^1^, it can guide policies that aim at concrete and impactful changes in the public health context.

## THE DYNAMICS OF INTERNATIONAL CLASSIFICATIONS OF DISEASES

With each ICD update, a variety of transformations can occur in the epidemiological profile of a region. The grouping of several diseases in a single pathology can increase the frequency of this disease as a cause of death, without its incidence necessarily increasing^2^.

Since the first classification in 1900, every 10 years new conferences were held for further revision. This dynamic occurred until the 10th revision (1989), when an interval of 30 years occurred for the presentation of the 11th version. This gap between revisions was only possible due to the adoption of annual updates policy. Thus, despite the long interval, updates of the 10th revision tried to make it less obsolete.

Nevertheless, limits and weaknesses of the ICD-10 eventually demanded a new revision. The main reasons were:

- Need for scientific update, incorporating more definitions and 41,000 more codes than the previous version. The increase in nosographic entities reflects the adaptation of classification to scientific development.
- Need for structural change to electronic format due to the introduction of electronic documentation in all areas of the health sector and in all work environments, including regions with limited resources. The new classification can be used online, or offline, where the stability of the internet is less reliable.
- Convenience of connection with other terminological systems – Family of International Classifications.
- Need to adapt to applications and obtain multilingual translations.
- Imposition to improve the reproducibility of important clinical details of the conditions, thus obtaining better usability – more clinical details with less training.
- Indispensability of enhanced user guidance. The enhancement in user orientation was based on pilot implementation projects.

ICD has been fundamental to the public and private health economy. In the financing of health services, the commonly used tool is the Inpatient Hospital Authorization (IHA), which is part of the diagnostic coding system of the classification. However, these forms are criticized because they are used predominantly for accounting purposes, disregarding the quality of coding and information. Nevertheless, these documents are presented as a rich source of administrative and epidemiological data, which can guide care and preventive actions. Therefore, we seek to advance in the critical analysis of the ICD uses.

The financial area of hospitals has always had greater attention to coding than the *Serviço de Arquivo Médico e Estatístic*a (SAME – Medical and Statistical Archive Service). The use of a classification as complete as ICD, for almost exclusively economic purposes, is an illogical underutilization. In this sense, facilitating the implementation and use of ICD-11 – which is achieved with digitization – as well as the broad and continued participation of several actors in the revision process, may be the first step to change the economic mindset of the classification.

Another interesting data is that, currently, 117 countries report causes of death to the WHO using ICD^3^. The classification supports the mapping of global mortality statistics; however, when evaluating morbidity, ICD does not receive the same importance in the international field.

## ICD-11

ICD-11 emerges in the context of a reality never experienced by societies. Before, it was almost impossible to integrate the whole world, but nowadays, considering the advent of computerized communication systems and the possibility of access, almost in real time, to relevant information, it is possible. ICD-11 was developed to reduce notification errors, to increase practicality and provide more scope to the information cataloged.

Some relevant points have guided this update. The wide range of professionals from different realities enabled a necessary heterogeneity to reflect regional particularities. Clinicians, statisticians, coders, information and technology specialists have integrated this update^3^, a type of global participation unprecedented in the history of ICD.

The new version is completely digital, aiming at practicality in registering and appointments, reducing notification errors and easing the dissemination and consolidation of this new version. ICD-11 brings changes to content and presentation format as well as new tools. Among the changes, the biggest advance is to accept suggestions from ICD users in the platform created for the revision. The 11th revision has a field implementation tool (ICD-FIT) with analytical capabilities that will allow continuous updates. The proposals will be analyzed by advisory groups and, if improvements are shown, they will be implemented in ICD-11. Thus, member States and WHO can assess the quality of coding and translations and improve the classification.

With these guidelines, ICD-11 presents great improvements compared with the previous version, among them^1^:

- Updated medical knowledge. Scientific development is represented by the wide range of nosographic entities.
- Contemporary concepts of primary care, with greater attention to the field in which most diagnoses are made.
- Revision and update of the section that deals with patient safety.
- Coding on bacterial resistance. A significant theme currently that was not contemplated in the previous version.
- Update of the HIV section, justified by the many findings on the subject in recent decades.
- Supplemental section for functional evaluation of the patient before and after the medical intervention.
- Incorporation of all rare diseases. Important gain in the field of scientific research.
- Codes referring to post-traumatic stress were updated and also simplified.
- Video game disorders have been added to the conditions that can generate addiction.

Other important nosographic and structural modifications of the new version of ICD:

- The new Chapter 7 deals with sleep-wake disorders, previously presented in the chapters on respiratory system, nervous system or mental health.
- Chapter 17, which addresses conditions related to sexual health, presents the condition “gender incongruity,” previously considered a “mental health” condition. The concern with social inclusion and acceptance of differences are relevant aspects of the new version of ICD. The codification of this condition by exclusively cultural thoughts, without proper scientific basis, would not be possible. This aspect can be indicated as one of the main points of improvement of ICD-11.
- The new Chapter 26 corresponds to the specific section on traditional medicine. In several countries, concepts and practices of traditional medicine are used without follow-up, due to lack of recording. With ICD-11, one can follow these practices and their effect on populations' health.

## IMPLEMENTATION OF ICD-11

The implementation of a complex and comprehensive coding system for classifying diseases requires great attention, considering the natural obstacles. According to the WHO, the transition from ICD-10 to ICD-11 should last from two to three years, and it may spend even greater time in locations with technological or logistical deficit^3^. Countries reporting to WHO should make this transition gradually, continuously and concomitantly with ICD-10.

The implementation of ICD-11 in Brazil will be a major challenge. Language is the initial unfavorable aspect. Portuguese is not an official language for the WHO, and the process of translation, adaptation, revision and implementation in a new language is a significant hampering and time-consuming aspect. The following project under development may be affected by the transition problems, since they use ICD-10 as a support base – Global Burden of Disease from Institute for Health Metrics and Evaluation (GBD/IHME); *Painel de Monitoramento da Mortalidade por Causas Básicas Inespecíficas ou Incompletas* (Garbage Codes); Analysis of Causes of (National) Death for Action (ANACONDA); and System for Automated Coding of Causes of Death and Selection of Underlying Cause of Death (IRIS).

However, it is essential to understand that the main challenge lies in the establishment of measures that change the understanding of ICD users. A consistent and complex classification system cannot be interpreted as a purely descriptor and bureaucratic document of morbidity and mortality conditions. The classification must be definitively consolidated in health practice as a strategic action capable of defining directions for the entire care and preventive system.

Since 2017, several countries – such as England, Wales, Scotland, Northern Ireland, Japan, Germany, and Australia – have participated in formative tests for the development of ICD-11. ICD-11 was nominated for adoption after being presented to the World Health Assembly in May 2019. In the 194 WHO member States, it will replace previous revisions from January 1st, 2022.

For the first time, American countries, along with the Pan American Health Organization, had the opportunity to participate in the early development stages of a new classification^1^. In addition to content collaboration, the participation also consisted in translating the ICD-11 into Spanish and pilot tests into English and Spanish. In the meantime, we also identified the main problems when analyzing the requirements for the transition to the new ICD version, such as lack of human and financial resources, insufficient number of trained coders, high turnover of the information team, occasional deficits in the infrastructure of information technology and need for wide dissemination.

The size and influence of the effective implementation of ICD-11 in developing countries have not been measured yet. Low- and middle-income countries account for a significant volume of diseases, while they have systems with limited funds for treatment, prevention and information gathering for health planning. In these countries, the concrete establishment of ICD-11 can ease the collection of care information and, consequently, generate better-founded health decision models.

The current version of ICD is available at the link: (https://icd.who.int/browse11/l-m/en).

## References

[1] 1. World Health Organization. ICD-11 for mortality and morbidity statistics. Version: 2019 April. Geneva: WHO; 2019 [cited 2019 Aug 20]. Available from: https://icd.who.int/browse11/l-m/en

[2] 2. Laurenti R, Buchalla CM, Mello Jorge, MHP, Lebrão ML, Gotlieb S. Perfil epidemiológico da saúde masculina na região das Américas: uma contribuição para o enfoque de gênero. São Paulo: Faculdade de Saúde Pública, Universidade de São Paulo; 1998.

[3] 3. World Health Organization. ICD-11 implementation or transition guide. Geneva: WHO; 2019 [cited 2019 Aug 20]. License: CC BY-NC-SA 3.0 IGO. Available from: https://icd.who.int/docs/ICD-11%20Implementation%20or%20Transition%20Guide_v105.pdf

