# Supplementary material for: International Classification of Diseases – 11th revision: from design to implementation
Source: Rev Saude Publica. 2020 Nov 4;54:104. doi: 10.11606/s1518-8787.2020054002120 (PMC7647465; doi:10.11606/s1518-8787.2020054002120)
Supplement: Translation

# Translation: Classificação Internacional das Doenças - 11ᵃ revisão: da concepção à implementação

**Authors:** Manuella Santos Carneiro Almeida, Luis Ferreira de Sousa, Patrícia Moreira Rabello, Bianca Marques Santiago

**Affiliations:** I João PessoaPB Brasil Universidade Federal da Paraíba. Programa de Pós-Graduação em Odontologia. Centro de Ciências da Saúde. João Pessoa, PB, Brasil; II João PessoaPB Brasil Hospital Estadual de Emergência e Trauma Senador Humberto Lucena. João Pessoa, PB, Brasil; III João PessoaPB Brasil Centro Odontológico de Estudos e Pesquisas. João Pessoa, PB, Brasil; IV João PessoaPB Brasil Universidade Federal da Paraíba. Departamento de Clínica e Odontologia Social. Centro de Ciências da Saúde. João Pessoa, PB, Brasil

**Keywords:** Classificação Internacional de Doenças, Organização Mundial da Saúde, Saúde Pública, Epidemiologia

## Abstract

A Organização Mundial da Saúde lançou em maio de 2019 a nova Classificação Internacional de Doenças (CID), 11ᵃ revisão. Como contribuição a essa transição, o objetivo deste texto é apresentar as principais mudanças da versão revisada da classificação e indicar os desafios mais prementes. Após 30 anos da publicação da CID-10, identificam-se desafios importantes quanto à nova classificação, que foi apresentada para adoção dos Estados-membros e entrará em vigor em janeiro de 2022. A finalidade da pré-visualização é permitir aos países planejar o uso e treinar seus profissionais. A nova versão é completamente digital, diminuindo assim os erros de notificação e facilitando a divulgação e consolidação da nova versão. A atualização deixa transparecer os avanços da compreensão cientifica e exige dos governos ações estruturantes e eficiência na implementação, para que todos que tratam da assistência possam se comunicar numa mesma linguagem, em escala mundial.

## INTRODUÇÃO

Diante do desenvolvimento social e econômico estabelecido e enfatizado pela globalização, as ações em saúde precisaram encontrar um novo enfoque de atuação. A incapacidade da assistência pontual e curativa em abranger ações com impacto realmente significativo para as necessidades sociais deixa clara a necessidade de uma visão assistencial mais complexa e planejada das ações em saúde pública. Assim, com o objetivo de minimizar afecções e gastos, é preciso antecipar ações frente às injúrias que acometem a sociedade.

Essa necessidade de planejamento das ações em prevenção e promoção de saúde alicerça uma coleta de dados cada vez mais fidedigna e planejada. Nesse contexto, segundo a Organização Mundial da Saúde (OMS)^1^, a Classificação Internacional de Doenças (CID) aparece como ferramenta fundamental para estabelecer políticas públicas alinhadas com as necessidades sociais.

A CID serve de base para identificar tendências estatísticas de saúde em todo o mundo. Como principal ferramenta de codificação dos agravos de mortalidade e morbidade^1^, ela pode nortear políticas que visam mudanças concretas e impactantes no contexto da saúde pública.

## A DINÂMICA DAS CLASSIFICAÇÕES INTERNACIONAIS DAS DOENÇAS

A cada atualização da CID, uma cascata de transformações pode ocorrer no perfil epidemiológico de uma região. O agrupamento de várias enfermidades em uma patologia pode aumentar a frequência desta enfermidade como causa de morte, sem que sua incidência tenha necessariamente aumentado^2^.

Desde a primeira classificação, em 1900, mais ou menos a cada 10 anos novas conferências eram realizadas para nova revisão. Essa dinâmica ocorreu até a 10ᵃ revisão (1989), quando houve, então, um intervalo de cerca de 30 anos para a apresentação da 11ᵃ versão. Esse grande intervalo entre as revisões só foi possível devido à adoção da política de atualizações anuais. Assim, apesar do grande intervalo, as atualizações da 10ᵃ revisão tentavam torná-la menos obsoleta.

Apesar disso, limites e insuficiências da CID-10 acabaram por demandar nova revisão. Os principais motivos foram:

- Necessidade de atualização científica, com incorporação de mais definições e 41 mil códigos a mais que a versão anterior. O aumento das entidades nosográficas reflete a adaptação da classificação ao desenvolvimento científico.
- Necessidade de mudança estrutural para formato eletrônico em virtude da introdução de documentação eletrônica em todas as áreas do setor da saúde e em todos os ambientes de trabalho, incluindo regiões com recursos limitados. A nova classificação pode ser usada on-line, ou off-line, onde a estabilidade da internet é menos confiável.
- Conveniência de conexão com outros sistemas terminológicos – Família das Classificações Internacionais.
- Necessidade de se adequar a aplicativos e obtenção de traduções multilíngues.
- Imposição de aprimorar a reprodutibilidade de detalhes clínicos importantes das afecções, obtendo-se, portanto, melhor usabilidade – mais detalhes clínicos com menos tempo de treinamento.
- Imprescindibilidade de orientação aprimorada ao usuário. As melhorias na orientação ao usuário tiveram base em projetos-piloto de implementação.

A CID vem sendo fundamental para a economia da saúde pública e privada. No financiamento dos serviços de saúde, a ferramenta comumente utilizada é a Autorização de Internação Hospitalar (AIH), que é parte do sistema de codificação de diagnósticos da classificação. Esses formulários, contudo, são objeto de crítica por serem utilizados predominantemente para fins contábeis, desconsiderando a qualidade da codificação e das informações. Apesar disso, esses documentos se apresentam como rica fonte de dados administrativos e epidemiológicos, que podem nortear ações assistenciais e preventivas. Busca-se, portanto, avançar na análise crítica dos usos da CID.

A área financeira dos hospitais sempre teve maior atenção à codificação do que o Serviço de Arquivo Médico e Estatística (Same). Utilizar uma classificação tão completa quanto a CID, para fins quase exclusivamente econômicos, é uma subutilização ilógica. Nesse sentido, facilitar a implementação e utilização da CID-11 – o que é conseguido com a digitalização –, bem como a participação ampla e continuada de diversos atores no processo de revisão, pode ser o primeiro passo para mudar a mentalidade econômica da classificação.

Outro dado de reflexão é que, atualmente, 117 países relatam causas de morte à OMS utilizando a CID^3^. A classificação contribui para o mapeamento das estatísticas mundiais de mortalidade; contudo, quando o agravo avaliado é de morbidade, a CID não recebe a mesma importância no campo internacional.

## A CID-11

A CID-11 surge no contexto de uma realidade nunca experimentada pelas sociedades. Em nenhum momento a integração mundial foi tão possível, graças ao advento dos sistemas informatizados de comunicação e da possibilidade de acesso, quase em tempo real, a informações relevantes. Para integrar-se a essa realidade, a CID-11 foi desenvolvida com a intenção de diminuir os erros de notificação, aumentar a praticidade e dar mais abrangência às informações catalogadas.

Alguns pontos relevantes nortearam essa atualização. A participação de profissionais de realidades distintas possibilitou uma heterogeneidade necessária para refletir as particularidades regionais. Clínicos, estatísticos, codificadores, especialistas em informação e tecnologia integraram a atualização^3^, em um tipo de participação global sem precedentes na história da CID.

Buscando por praticidade de registro e consulta, a nova versão é completamente digital, diminuindo assim os erros de notificação e facilitando a divulgação e consolidação da nova versão. A CID-11 traz mudanças de conteúdo e formato de apresentação e novas ferramentas. Dentre as alterações, o maior avanço é aceitar sugestões dos usuários da CID por meio da plataforma criada para a revisão. A 11ᵃ revisão apresenta uma ferramenta de avaliação de implementação (CID-Fit) com recursos analíticos que permitirão a atualização contínua da plataforma. As propostas serão analisadas por grupos consultivos e, caso se mostrem melhorias, serão implementadas à CID-11. Assim, os Estados-membros e a OMS podem avaliar a qualidade da codificação e das traduções e melhorar a classificação.

Com esses norteadores, a CID-11 apresenta melhorias notórias em comparação com a versão anterior, dentre elas^1^:

- Conhecimento médico atualizado. A ampla gama de entidades nosográficas reflete o desenvolvimento científico.
- Conceitos contemporâneos de atenção primária, com maior atenção ao campo de atuação em que grande parte dos diagnósticos são realizados.
- Revisão e atualização da seção que versa sobre a segurança do paciente.
- Codificação sobre resistência bacteriana. Tema significativo atualmente e que não era contemplado na versão anterior.
- Atualização da seção sobre HIV, justificada pelos muitos achados sobre o tema nas últimas décadas.
- Seção suplementar para avaliação funcional do paciente antes e após a intervenção.
- Incorporação de todas as doenças raras. Ganho importante no campo da pesquisa científica.
- Os códigos referentes a estresse pós-traumático foram atualizados e simplificados.
- Os transtornos dos jogos eletrônicos foram adicionados às condições que podem gerar adição.

Outras modificações nosográficas e estruturais importantes da nova versão da CID:

- O novo Capítulo 7 trata de distúrbios de sono-vigília, anteriormente apresentados nos capítulos sobre sistema respiratório, sistema nervoso ou saúde mental.
- O Capítulo 17, que aborda as condições relacionadas à saúde sexual, apresenta a condição “incongruência de gênero”, antes considerada afecção de “saúde mental”. A preocupação com a inclusão social e a aceitação das diferenças são aspectos relevantes da nova versão da CID. A codificação dessa condição por meio de pensamentos de cunho exclusivamente cultural, sem o devido embasamento científico, não seria possível. Esse aspecto pode ser apontado como um dos principais pontos de melhoria da CID-11.
- O novo Capítulo 26 corresponde à seção específica sobre medicina tradicional. Em vários países, utilizam-se conceitos e práticas da medicina tradicional sem acompanhamento, devido à falta de registro. Com a CID-11, pode-se acompanhar essas práticas e seu impacto sobre a saúde das populações.

## IMPLEMENTAÇÃO DA CID-11

A implantação de um sistema de codificação complexo e abrangente de classificação de doenças demanda grande atenção, dados os obstáculos naturais. Segundo a OMS, a transição da CID-10 para a CID-11 deve durar de 2 a 3 anos, podendo despender um tempo ainda maior em localidades com déficit tecnológico ou de logística^3^. Os países que se reportam à OMS devem fazer essa transição de forma paulatina, continuada e concomitante com a utilização da CID-10.

A implementação da CID-11 no Brasil será um grande desafio. O aspecto desfavorável inicial é o idioma. O português não é língua oficial para a OMS, e o processo de tradução, adaptação, revisão e implementação na nova língua é um dificultador importante, que demandará tempo. Outro agravante são os projetos que estão em desenvolvimento e têm o CID-10 como base de apoio – *Global Burden of Disease from Institute for Health Metrics and Evaluation* (GBD/IHME); Painel de Monitoramento da Mortalidade por Causas Básicas Inespecíficas ou Incompletas *(Garbage Codes)*; Análise de Causas de Morte (Nacional) para Ação (Anaconda); e Sistema para Codificação Automatizada de Causas de Morte e Seleção da Causa Básica de Morte (Iris).

Porém, é fundamental entender que o principal desafio está no estabelecimento de medidas que mudem o entendimento dos usuários da CID. Um sistema tão consistente e complexo de classificação não pode ser visto como um documento puramente descritor e burocrático de condições de morbidade e mortalidade. A classificação precisa ser definitivamente consolidada na prática de saúde como uma ação estratégica capaz de definir rumos para todo o sistema assistencial e preventivo.

Desde 2017, vários países – como Inglaterra, País de Gales, Escócia, Irlanda do Norte, Japão, Alemanha e Austrália – têm participado de testes formativos para o desenvolvimento da CID-11. Após a apresentação à Assembleia Mundial da Saúde em maio de 2019, a CID-11 foi indicada para adoção. Nos 194 Estados-membros da OMS, ela substituirá as revisões anteriores a partir de 1 de janeiro de 2022.

Pela primeira vez, os países das Américas, por meio da Organização Pan-Americana da Saúde, tiveram a oportunidade de participar dos estágios iniciais de desenvolvimento de uma nova classificação^1^. Além da colaboração de conteúdo, a participação consistiu também na tradução da CID-11 para o espanhol e testes-piloto em inglês e espanhol. Nesse ínterim, no estudo de requisitos para a transição à nova versão da CID, também foram identificados os principais problemas, como falta de recursos humanos e financeiros, número insuficiente de codificadores treinados, alta rotatividade da equipe de informações, déficits ocasionais de infraestrutura de tecnologia da informação e necessidade de ampla divulgação.

A dimensão e a influência da implantação efetiva da CID-11 em países em desenvolvimento ainda não foram mensuradas. Os países de baixa e média renda respondem por um volume significativo de doenças, ao mesmo tempo em que têm sistemas com verbas limitadas para tratamento, prevenção e coleta de informações para o planejamento em saúde. Nesses países, o estabelecimento concreto da CID-11 pode facilitar a coleta de informações assistenciais e, consequentemente, gerar modelos de decisão em saúde mais bem fundamentados.

A versão atual da CID está disponível no link: (https://icd.who.int/browse11/l-m/en).
